# “The forest and the trees”: a narrative medicine curriculum by residents for residents

**DOI:** 10.1038/s41390-024-03142-2

**Published:** 2024-03-22

**Authors:** Anoushka Sinha, Carly S. Slater, Alyson Lee, Harini Sridhar, Deepthiman Gowda

**Affiliations:** 1 Department of Pediatrics, University of California, San Francisco, San Francisco, CA USA; 2 Department of Pediatrics, NewYork-Presbyterian Morgan Stanley Children’s Hospital/Columbia University Irving Medical Center, New York, NY USA; 3 The University of Pittsburgh School of Medicine, Pittsburgh, PA USA; 4 The University of North Carolina School of Medicine, Chapel Hill, NC USA; 5 Kaiser Permanente Bernard J. Tyson School of Medicine, Pasadena, CA USA

## Abstract

**Abstract:**

A 7-session narrative medicine (NM) curriculum was designed and facilitated by pediatrics residents for pediatrics residents in order to unpack challenging experiences during clinical training and strengthen relationships with colleagues and patients. The primary facilitator, a resident with a master’s degree in NM, provided facilitator training to her co-residents with whom she co-led the workshops in the curriculum. We conducted, transcribed, and analyzed individual interviews of 15 residents, with three resultant themes: reflection on personal and professional identity; connection to others and community building; and reconceptualization of medical practice. Residents shared that they experienced greater solidarity, professional fulfillment, appreciation for multiple facets of their identities, recognition of holding space for vulnerability, and advocacy for marginalized populations. Our study highlights the feasibility and effectiveness of peer-led NM workshops to enhance clinical training through self-reflection, inclusion of persons from underrecognized backgrounds, and promotion of values consistent with humanistic care.

**Impact:**

- A novel narrative medicine curriculum was designed and facilitated by pediatrics residents for pediatrics residents.
- The curriculum was feasible and acceptable to pediatrics residents and required a facilitator with content and methodology expertise in narrative medicine to train additional facilitators.
- Three themes emerged from resident interviews: reflection on personal and professional identity; connection to others and community building; and reconceptualization of medical practice on individual and global levels.

## Introduction

In “A Plea for Narrative Medicine in Pediatric Residency Training,” Diorio and Nowaczyk argue for the integration of narrative medicine (NM) into pediatrics residency programs given the tragedy, stress, and isolation routinely encountered in training.^1^ Their plea took on new weight in the covid-19 era, when healthcare professionals faced greater responsibilities amid tremendous loss and a sharp rise in burnout, particularly for resident physicians.^2,3^ The pandemic also highlighted healthcare disparities, police brutality, and violence in communities of color.^4–6^ This exposure led to a critical examination of medical culture and an exploration of how healthcare professionals, pediatricians in particular, can discuss anxiety and loss and regain fulfillment in their work.^7,8^ NM is a branch of health humanities, with its conceptual framework of attention, representation, and affiliation,^9^ that seeks to create opportunities for reflection, group cohesion, and development of skills of close listening amongst those engaged in delivering healthcare.

Peer teaching and support are increasingly valued in medical education.^10,11^ Studies have demonstrated that physicians often identify peers as the most acceptable sources of support,^12,13^ and that resident wellness in particular benefits from peer-support interventions.^14–16^ Trainees value learning from peers due to their social and cognitive congruence, creating a climate of safety and comfort in which learners can be more vulnerable with each other.^10,17,18^ Given the recognized benefits of peer education and support, and the relative shortage of trained NM facilitators, we designed and implemented a resident-led NM curriculum to be implemented specifically for resident physicians.

This paper describes an evaluation of the first NM curriculum designed and facilitated by pediatrics residents for pediatrics residents, with the aim of deepening the experience of affiliation to oneself, to others, and to the medical profession and world at large. This model enabled a curriculum that could attend to the experiences of trainees during and beyond the covid-19 healthcare crisis and address relevant healthcare barriers and biases with the goal of improving care for all.

## Methods

### Qualitative approach

In this study, we worked within an interpretivism approach, focusing on understanding the subjective experience of NM workshops for pediatrics residents.^19^ We applied the principles of codebook thematic analysis to data in the form of interview transcripts to generate themes.

### Workshop framework and participants

An NM curriculum of seven workshops for residents of all training years was integrated into the pediatrics residency program at New York-Presbyterian Morgan Stanley Children’s Hospital at Columbia University Irving Medical Center (CUIMC). The curriculum was guided by the conceptual framework of attention, representation, and affiliation. The peer educational model was implemented to reduce hierarchies between NM workshop facilitators and participants while leveraging healthy interdependency amongst residents to support one another in dialog involving challenging and potentially polarizing subjects.

Workshops took place every 6 weeks from August 2020 to June 2021 as part of required ACGME noontime didactic hours. Urgent clinical duties took precedence over didactics; thus, not all residents could participate in each session. Author AS, a graduate of Columbia University’s NM Master’s Program, conducted one-on-one basic NM facilitator training for three other residents who volunteered to co-lead the workshops, and none of whom had prior NM training. This training entailed a 30-min meeting prior to each workshop (for a total of 3.5 h of facilitator development) reviewing the principles of NM, the workshop structure, facilitation techniques, and a review of texts and prompts to be used in the workshop. AS co-facilitated each workshop with one other resident, encouraging them to assume more leadership of the sessions as the curriculum progressed. Some resident participants had prior NM exposure, but none besides AS had formal NM training. Each workshop was held in a hybrid (in-person and remote participation using Zoom) format due to pandemic limitations with 15-30 residents attending each session out of a total of 75 residents across all years of the residency program. The majority of participants attended in person, with 2–5 participants joining remotely for each session. The sessions included a mix of residents from across the three years of training. Generally, two facilitators were utilized for each workshop session for the hybrid design, with one seated in a circular chair arrangement with resident participants and another situated in the room outside the circle, engaging with the remote audience. After each workshop, AS and co-facilitators debriefed about the workshops, which in turn informed future sessions.

### Session description

Workshops followed the methodology developed by the NM Program at Columbia University.^20^ Each 1-h workshop consisted of three activities: 30 min of close reading or observation of a creative work (e.g., a poem, novel/short story excerpt, visual art, comic, or film segment); 5 min of writing to a prompt; and 15 min of sharing written responses to the prompt in dyads, followed by sharing with the whole group. The curriculum (including choice of creative works and prompts used) was dynamically adapted to suit residents’ needs, with works centering on mortality, illness, relationships, medical training, and current events (including the Black Lives Matter movement, violence against Asian Americans, and bias towards transgender and nonbinary patients) (Table 1). The themes contained in the creative works and prompts were chosen by the facilitators based on challenges they felt residents were facing in training that may benefit from the NM workshop method.

**Table 1:** Workshop Texts and Writing Prompts.

Piece 1	Piece 2	Prompt
“An Intern’s Recollection of a Night at the VA, July 2004” by Doug Hester	“Starfish” by Mary Oliver	Write about learning to love what isn’t easy.
“Rainy Day, Boston” by Childe Hasam	“The Raincoat” by Ada Limón	Write about your raincoat.
“The Thankful Poor” by Henry Ossawa Tanner	“Thanks” by W.S. Merwin	Write about something or someone you are thankful for.
Graphic Medicine Pieces by Brandon Mogrovejo (PGY1)		Draw four panels that capture an important experience in your training.
“Delayed” by R. Kikuo Johnson	“Everything” by Jane Wong	Choose one of the characters in this piece. Imagine and write from their perspective.
“Blue Monday” by Annie Lee	“Dust” by Dorianne Laux	Write about a message you were too tired to receive in the moment.
“Queer in Common Country” by Kara Sievewright		Write about a time when you witnessed or experienced LQBTQ+ sensitive (or insensitive) care.

### Data collection

Semi-structured interviews consisted of nine standardized open-ended interview questions accompanied by probing inquiries. These questions explored how the NM workshops impacted residents’ experiences inside and outside the hospital, their relationships with patients, colleagues, and loved ones, as well as the experience of facilitation by co-residents (Table 2). The interviews were conducted by authors (HS and AL) who were not involved in the pediatrics residency program in an effort to allow residents to speak more freely about the workshops. Interviews were conducted over Zoom for 30–60 min and were transcribed with Temi, an automatic transcription service. The study was approved by the International Review Board at CUIMC (IRB-AAAS4136).

**Table 2:** Interview Guide.

Interview Questions
1. What was participating in the narrative medicine workshops like for you?
2. What was being involved in the narrative medicine workshops like for you in the context of the pandemic? a. Did any issues related to COVID come up in your or other’s writings? b. How about in the discussions? If yes, can you tell me more?
3. What did you take away from the experience of these workshops? a. What impact did this work have on your work life? What impact did this work have on your life outside of work? b. Is there anything you learned or practiced in these sessions that you plan on continuing?
4. How do you think these narrative medicine sessions have impacted: a. Your relationships with colleagues, if at all? b. Your relationships with patients, if at all? c. Your own reading, writing, and creative interests or habits?
5. What was it like to have the workshops facilitated by co-residents? a. If applicable, what was the co-facilitation like for you?
6. What does it mean to you to have NM workshops as part of our dedicated noon conference curriculum? a. Do narrative medicine workshops belong in a pediatric residency program? Why or why not?
7. What challenges did you face, if any, when deciding to attend or engage with the workshops?
8. Do you have any advice for future workshops?
9. Were there any topics/questions that we missed during this session that you think were important?

Interviews were voluntary, and participants who had attended at least two workshops were recruited by purposive sampling. In total, 15 residents (5 residents from each class) were interviewed over Zoom, constituting 20% of the residency program. All identifying information aside from participants’ level of training was excluded from the transcripts.

### Qualitative data analysis

AS, AL, HS, and CS used Dedoose software to create descriptive codes, compile a codebook, and iteratively code all transcripts together to maintain intercoder reliability. The codebook was initially built upon the analysis of one transcript. The team then applied codes across subsequent transcripts, modifying existing codes and adding new ones to the codebook in the process. Codes conveying related concepts were clustered into categories, which were then clustered into higher-order themes. While many codes were tied to the specific language used by participants, others were identified by associated NM principles. Such codes included bearing witness,^21^ narrative humility,^22^ cultural humility,^23^ and attentive listening.^20^ A final pass of coding through all transcripts was done once the codebook was finalized. The team kept an audit trail documenting observations and conclusions from weekly meetings.

## Results

### Reflection on personal and professional identity

Participants described that the NM workshops prompted a re-evaluation of their identities as physicians and allowed them to develop reflective skills to navigate professional and personal spheres. Many residents acknowledged how they often compartmentalized their physician identities from the rest of their lives and how NM allowed them to reconcile these multiple identities. As one PGY1 shared:

We were able to reflect a little bit about how our personal relationships are not completely different from the relationships that we view and experience here in the hospital…I think we compartmentalize what we’re doing in medicine as a separate activity. But really, it’s in sync with our lives. They’re very much interconnected.

Others shared how thinking “through a narrative lens” brought them back to when they first started medical school, reorienting them to perspectives they had prior to medical training. One PGY2 said:

[NM] brings me back to when I was a new medical student…I think there’s this time in medical school where you’re starting out and you’re like, ‘Oh, of course I’m always going to feel the perspective of the patient. I’m never going to get caught up in the day-to-day of a hospital. This is why I went into medicine.’ And it just happens when you’re busy, when you’re tired, when being a doctor gets so much a part of your identity and your brain and the way that you think, that you do [get caught up].

In essence, residents described that NM allowed them to reconnect to other parts of themselves and bring these roles into their lives as physicians.

By bringing their whole selves into conversation with their identities as doctors, residents expressed how workshops served as a “space of rest” and led them to rediscover former creative interests that had been abandoned during residency. One PGY2 said: “[NM] really re-emphasized my creative interests…It gives me an outlet where I can…do things outside of work that restore me, and then go back to work with that sense of openness to whatever I encounter in the day to day.”

Residents acknowledged that the sessions became a part of honing their wellness and that by taking better care of themselves, they could be more present for their patients and better physicians overall. As one PGY3 shared, “I probably was a happier person after [NM]…[I] didn’t need as much time to recharge when I would come home after work.”

### Connection to others and community building

As highlighted by the most frequently used code, “reinforcing relationships,” residents shared that the workshops helped them deepen relationships with colleagues, patients, and others outside of work, thereby mitigating the isolation that often accompanies medical training. Participants described “a loss of community” during the covid-19 pandemic and that the workshops offered “a little piece of that back.”

Participants commented on the power of sharing their experiences with co-residents, often discovering new aspects of their lives. One PGY1 said, “I learned a lot about where my co-residents are coming from themselves…it made me really proud to be a resident here and [hear] how thoughtful and caring and patient-centred all my co-residents are…It was really enlightening and made me feel really connected to them.” Workshops also offered opportunities for kinship between residency classes. One PGY1 shared:

“After [a workshop] I had a pretty long conversation with a resident who was a year above me…It was just a nice connection that I wouldn’t have made otherwise. [NM] gave us a safe space to have that connection. So especially amongst people not in my class, it has been helpful to building bridges.

Indeed, many residents spoke to the meaningfulness of a space created by residents, for residents. One PGY2 articulated that “resident-led things, they’re not that common…[I]t’s one of the paradigms that residents need to take charge of educating one another and taking care of one another more. [NM] is a great example of a place where that happens.” Others corroborated this point by sharing how the dynamic would have changed had the sessions been faculty-driven; a PGY3 stated:

If it was coming from faculty, it would be different. I think people would be less open to expressing themselves openly and honestly. It makes you want to participate because you know that [the residents] put the work into setting up this session and they’ve been very thoughtful about what the goals of the session are. You just want to show up for your co-resident.

Thus, residents spoke to the sanctity of the resident-led space and how this allowed deeper intimacy to form while inspiring a sense of fraternal responsibility to one another.

One dividend of this growing camaraderie was how it normalized experiences and emotions during residency. One PGY1 commented:

As an intern, you feel super alone a lot of the time, because it’s all very new and you do not really know what is quote-unquote “normal” to feel during this. Hearing my co-residents talk about their experiences and their feelings throughout their time has…made me feel more confident and validated as a physician. I feel less alone and more like one of the group. I guess it’s just made me feel more confident as a physician towards patients.

Participants thus highlighted the impact that sharing can have, not only to build stronger bonds as a cohort but also to soothe professional anxieties about how they should or should not be experiencing residency.

Residents also described how workshops facilitated a sense of communion between themselves and patients by encouraging them to step back from their perspectives as trainees and instead consider the family in front of them. One PGY2 said:

You think about what you’re treating and about what you’re managing when you enter the room. But then, it’s great to have that perspective of sometimes just going into the room and seeing like, “wow, this is a family. I see these toys here.” I can think about what that family unit is just outside of the hospital, and how I can help them get back to that.

In this sense, workshops served as a pause for residents to remember their purpose as caregivers and prioritize their patient’s humanity before focusing on medical decision-making. Another resident expressed that finding that pause allowed for recognising the beauty of the clinical encounter: “So many beautiful things that happen in these patient encounters that…you don’t always get to appreciate when you’re in the weeds. But it pulls you back to see the forest and the trees.”

Residents also reflected on how centering the patient perspective and engaging in literature led to tangible changes in their clinical practice. One PGY3 alluded to a story from one workshop which was written from a patient’s perspective:

Being able to see things from the voice and perspective of a patient really helps me self-evaluate how I am delivering news…It helped me be a lot more conscious of the kind of words that I used, the way that I express myself because things that initially appear harmless looking at it from the patient’s perspective, through the literature, it can be received in a very different way. And [NM] helps shed light on that and helps me change some of my approaches.

Another PGY1 reflected specifically on how a workshop featuring a comic about a transgender patient’s clinic experience impacted their understanding of patients from marginalized backgrounds. This discussion stirred a deeper consideration of the experience of being misgendered: “It was like a particular switch to further reflect on how language that we use is so, so important. And I try to be conscious with every patient I see, to make sure that I’m communicating the best way I can.” Residents expressed a greater understanding of what it means to advocate for marginalized patients and were motivated to educate themselves and “provide better, more comprehensive care.” In tandem, residents expressed that the workshops led to an emphasis on relationship-building and gave them new tools for fostering stronger relationships both with their peers and with patients.

### Reconceptualization of medical practice

Finally, workshops prompted residents to reflect critically on their place within and outside of medical culture. Residents often associate medical culture with an emphasis on the biomedical sciences over biopsychosocial approaches, pressure to limit emotions in the workplace, hierarchy, perfectionism, and fear of failure. They repeatedly characterized NM workshops as a form of “nontraditional medical education” that taught “skills that you can’t teach in a book.” They praised the “different framing” of NM and saw it as a contrast to the “overmedicalization of patients” that they often received from other conferences.

In particular, residents shared that they used the workshops as a space to unpack emotions often discouraged in the workplace. One PGY2 said, “In medicine…you’re not supposed to feel, you’re not supposed to express emotion. That’s supposed to be separate…People may go so far as to [say] that that was a sign of being an incompetent physician.” This resident went on to share how the “safe space” of NM created a new culture around emotionality, where vulnerability and naming emotions “makes you a stronger person.”

For many who self-identified as more private individuals, this led to tangible shifts in their approach towards vulnerability in the workplace; one PGY3 said:

During the first workshop, I don’t think I talked very much because of that feeling of wanting to compartmentalize my emotions about work…I wouldn’t say that I became a super sharer by the end, by any means, but definitely I think I became more open to realizing that…it is okay to share with people…some of the frustrations I might be having, or if I’m feeling overall discouraged.

This culture of emotional openness led some residents to even reconceptualize their idea of failure: “I feel like more of my experiences are just in being a physician or a resident and not necessarily failures on my end.” By engaging with their peers on an emotional level, residents were able to recognize that emotions like shame or grief are not signifiers of failure but normal responses to tragic circumstances.

For others, NM workshops gave them the necessary space to consider their role in patients’ lives. One PGY3 said, “You are going through these very traumatic, intimate experiences…And you become this interloper in people’s dramatic points in their lives. So I definitely look at it as a privilege, which I think in residency I’ve grown to recognize more and more…that’s only enhanced within the [NM] workshop.” Recognizing their privilege and responsibility as physicians served as a buffer to the more traditional, antiquated power differential between patients and physicians.

Similarly, others emphasized how NM helped them see the value of learning from patients, flipping the traditional hierarchy:

It further elucidates that [patients] are just other people like us. That might seem very obvious. But sometimes when you go through different academic exercises to try to figure out what’s going on with patients, you’re more focused on that than the patients’ experiences themselves. [NM] sessions…show how our experiences are very much the same. And something might happen tomorrow that might put me in a position that’s the exact same as the patient I’m taking care of.

Thus, NM facilitated a needful perspective shift, bringing residents out of their gaze as medical practitioners and back to their core humanity. Residents described that the workshops prompted them to orient towards patients from their shared humanity rather than their position of power. As one PGY2 said, “[We worked] this other part of my brain and also my soul that we don’t typically get to do in the hospital.”

## Discussion

This is the first evaluation of an NM workshop designed and facilitated by residents for residents. Prior literature suggests peer education nurtures spaces where vulnerability is more feasible and where challenging topics may be explored,^10,17,18^ a point substantiated by resident responses to our curriculum. Creating such a climate is crucial to achieving the tenets of NM to promote attention, representation, and affiliation to oneself and one’s community. This NM curriculum that utilized peer design and facilitation was found to be feasible and effective for resident education.

Residents recognized that the content and outcomes of the workshops could be perceived as contrary to the standards of biomedical culture, which classically dictate that physicians should compartmentalize their emotions at work.^24^ This reversal of tradition afforded by NM has historically been impeded by resistance within medical institutions posed by both students and faculty.^25^ By introducing a curriculum by residents for residents, our intervention represents a model for NM to be adapted in a variety of educational and clinical settings that facilitates this culture shift through the power, investment, and interdependency of a peer-based collective. This premise aligns with prior literature demonstrating that peer education can transform a training program’s community of practice, with positive changes to its culture and social capital.^26^

Through the NM workshops, residents were able to appreciate that being a doctor does not need to be divorced from all other parts of the self but can rather be supported by them; that lived experience outside of the hospital and the emotional acuity often cultivated with those experiences can enhance the role of a physician; that being a doctor does not center exclusively on clinical acumen but also on understanding social determinants of health, sensitivity to bias, and the value of advocacy; and that medicine’s definition of what makes a doctor is evolving and inclusive of gentler, more vulnerable, and more complex ways of being.

We acknowledge that residents who were open to sharing their experiences during the interviews may be particularly drawn to the health humanities. In addition, our qualitative data comes from one source: individual interviews of residents; data can be complemented in future studies by facilitators’ field notes as well as interviews with stakeholders such as faculty members who work with residents to further evaluate the impact of NM in clinical settings. Finally, this intervention was led by a resident facilitator with a master’s degree in NM who provided basic training to resident co-facilitators. Master’s training provides robust preparation for developing and implementing such curricula. However, we feel that opportunities short of a master’s degree (e.g., weekend workshops in narrative medicine and certificates of professional achievement, both offered at Columbia University, in addition to facilitator training programs) may allow dissemination of this model to other settings. Further evaluation of such efforts is needed to better understand optimal dissemination.

Residency training entails many personal and professional challenges, and our study demonstrates that an NM curriculum by residents for residents is a promising application of peer-led education to promote reflection, close listening, and solidarity. Such a curriculum may be of particular benefit to pediatricians and pediatricians-in-training, who attend to children’s stories across the span of development and within the context of their families and communities. Indeed, in the words of one resident, NM can clear some space to see “the forest and the trees.”

## Data Availability

The datasets generated during and/or analyzed during the current study are available from the corresponding author upon reasonable request.
